# ASQ-3 and BSID-III’s concurrent validity and predictive ability of cognitive outcome at 5 years

**DOI:** 10.1038/s41390-023-02528-y

**Published:** 2023-02-25

**Authors:** Cian Duggan, Alan D. Irvine, Jonathan O’B Hourihane, Mairead E. Kiely, Deirdre M. Murray

**Affiliations:** 1 Department of Paediatrics and Child Health, Cork University Hospital, Cork, Ireland; 2 Department of Clinical Medicine, Trinity College, Dublin, Ireland; 3 Department of Paediatric Dermatology, Our Lady’s Children’s Hospital, Dublin, Ireland; 4 The INFANT Research Centre, University College Cork, Cork, Ireland; 5 University College Cork, Cork Centre for Vitamin D and Nutrition Research, School of Food and Nutritional Sciences, Cork, Ireland

## Abstract

**Background:**

Early detection of cognitive disability is challenging. We assessed the domain-specific, concurrent validity of the ages and stages questionnaire (ASQ-3) and the Bayley Scales of Infant and Toddler Development (BSID-III), and their ability to predict cognitive delay at school age.

**Methods:**

Within a longitudinal birth cohort study, a nested cohort of children was assessed using ASQ-3 and BSID-III at 24 months, and at 5 years using the Kaufmann brief IQ test (KBIT).

**Results:**

278 children were assessed using BSID-III and ASQ-3 at 24-months; mean(SD) BW = 3445(506) grams, M:F ratio=52:48. ASQ-3 had reasonable predictive ability (AUROC, *p* value, sensitivity:specificity) of same domain delay for motor (0.630, *p* = 0.008, 50%:76.1%) and language (0.623, *p* = 0.010, 25%:99.5%) at 2 years, but poor ability to detect cognitive delay compared to BSID-III (0.587, *p* = 0.124, 20.7%/96.8%;). 204/278 children were assessed at 5 years. BSID-III language and cognition domains showed better correlation with verbal and nonverbal IQ (R = 0.435, *p* < 0.001 and 0.388, *p* < 0.001 respectively). Both assessments showed high specificity and low sensitivity for predicting delay at 5 years.

**Conclusions:**

The ASQ-3 cognitive domain showed poor concurrent validity with BSID-III cognitive score. Both ASQ-3 and BSID-III at 2 years poorly predict cognitive delay at 5 years.

**Impact:**

- The ASQ-3 does not adequately detect cognitive delay or predict cognitive delay at 5 years, particularly for children with mild to moderate delay.
- The ASQ-3 shows reasonable concurrent validity with the motor and language subscales of the BSID-III. Neither early screening nor formal developmental testing demonstrated significant predictive validity to screen for cognitive delay at school age.
- This article highlights the need to analyse our existing model of using the ASQ-3 to screen for cognitive delay in children aged 2 years.

## Introduction

Early intellectual function is a key predictor of adult health and well-being.^1^ Pre-school identification of those most at risk allows for early intervention which improves developmental outcomes as demonstrated in a Cochrane review (2015) and by refs. ^2,3^ Current assessment methods rely on parental report or structured direct assessments based on the ascertainment of developmental milestones. These developmental milestones are, in themselves, approximate metrics for later cognition. The ICD-11 defines neurodevelopmental disorders as *“behavioural and cognitive disorders that arise during the developmental period that involve significant difficulties in the acquisition and execution of specific intellectual, motor, or social functions”.*^4,5^ The prevalence of neurodevelopmental delay has been reported as high as fifteen to eighteen percent, with approximately three percent having severe delay.^6–8^ Incidence increases significantly in high risk groups such as small for gestational age^9^ and preterm infants ranging from 50.2–62.5% in those born <29 weeks’ gestation.^10,11^ The American Academy of Paediatrics recommends periodic screening using a validated tool at nine, eighteen and thirty months of age to allow targeted early intervention.^12^

The Ages and Stages Questionnaire 3 (ASQ-3) is a parentally completed developmental screening tool which can be performed in 10–15 minutes^5^ helping to identify children requiring further developmental evaluation.^8^ The Bayley Scales of Infant and Toddler Development III (BSID-III) is a more comprehensive developmental assessment undertaken by a trained healthcare professional. These tools are further described in the methods section. Together, these tools comprise a valuable resource in clinical practice, however, there is a paucity of research, with often contradictory views on their validity, agreement and predictive value for later cognitive outcome. One systematic review identified three papers which assessed the ASQ-3’s validity in conjunction with the BSID-III (the accepted gold standard at this age).^13–16^ The ASQ-3 self-reports a sensitivity of 86% and a specificity of 85%.^5^ However, independent studies report sensitivity and specificity ranging from 67–100% and 65–93%, respectively^17–21^ when compared to the BSID-III (as a gold standard). Velikonja et al. indicated that the lack of research and quality of studies made it difficult to draw any clear conclusions on sensitivities nor specificities of the ASQ-3.^16^ The ASQ has been demonstrated to have a higher sensitivity in high-risk groups, particularly for those with severe delay. Unfortunately, the rate of detection is markedly lower for mild and moderate delay, a group with a much higher prevalence in the general population.^7,18,19^ As many countries are using the ASQ-3 as the standard developmental screening tool for healthy population screening, it is important to establish its validity and reliability for both detecting developmental and cognitive delay in addition to predicting outcomes at school age.

The BSID-III is equally not without its own confounding factors. Ten percent of the cohort used for establishing normative ranges for each of its three domains; motor, verbal and non-verbal; was comprised of high-risk infants including children with trisomy 21, cerebral palsy, pervasive developmental disorder, premature birth, specific language impairment, prenatal alcohol exposure, birth asphyxiation, small for gestational age.^22^ By including high risk infants in the cohort developing the BSID-III, the scores in each domain is right skewed which lowers cut-off values for delay. This increases the likelihood of children with mild to moderate delay remaining undetected by this tool. The ability of the BSID-III to detect intellectual disability has been questioned.^23^ This study aimed to assess the domain specific, concurrent validity of the ASQ-3 to predict cognitive delay as detected by the BSID-III at 24 months. We also aimed to assess the predictive ability of both the ASQ-3 and the BSID-III to predict cognitive outcome at school age.

## Methods

This study was a secondary data analysis of a nested cohort of the Cork BASELINE Birth Cohort Study.^24^ Participants were recruited from the Cork BASELINE Birth Cohort Study born between March 2009 and September 2011. BASELINE (http://www.baselinestudy.net) was established in 2008 as a follow-up to the Screening for Pregnancy Endpoints (SCOPE) pregnancy study. The study participants, aims and methods of the Cork BASELINE birth cohort study have been previously reported.^25–28^ Ethical approval for the Cork BASELINE Birth Cohort Study was granted by the Clinical Research Ethics Committee of the Cork Teaching Hospitals (Ref: ECM 5 (9) 01/ 07/2008), and the study is registered (Ref: NCT01498965) with the United States National Institutes of Health Clinical Trials Registry (http://www.clinicaltrials.gov).

Within the birth cohort, a nested cohort of children who were small or thin for gestational age^28^ (SGA and TGA) were invited for additional assessments using the BSID-III at 24 months. TGA refers to infants with a body fat mass <10th percentile. The outcome of these children and their sex and age matched controls has been previously described.^28^ Controls were born at term and had birth weights which were appropriate for gestational age (AGA). All BASELINE cohort participants were invited to attend for an IQ assessment at 5 years using the KBIT.

Three developmental assessment tools were utilised in this study; the ASQ-3, BSID-III and KBIT. The Ages and Stages Questionnaire 3 (ASQ-3) is a parentally completed screening tool which can be performed in 10–15 minutes assessing the domains of communication, gross motor, fine motor, problem solving, and personal-social at a variety of ages and in multiple languages.^5^ It aims to identify children not achieving age-appropriate developmental milestones across these domains and subsequently refer them for further assessment. It’s simplicity and short completion time allow for quick evaluation and identification of children requiring further developmental evaluation.^8^ The BSID-III is undertaken by a trained healthcare professional over one to two sessions requiring direct observation of skills in the domains of cognition, language, social-emotional, motor and adaptive behaviour. It is used to identify children with developmental delay who may require intervention services. The KBIT is a professionally administered intelligence quotient assessment which measures verbal and non-verbal intelligence from the ages of 4–90 years.

The BSID-III assessments were administered by research psychologists trained in BSID-III. ASQ-3 questionnaires were posted to the parents the week prior to assessment and they were asked to complete in advance. Any queries were answered on the day of assessment. KBIT was performed by a research nurse trained in the administration of the test. Parents and caregivers were contacted by telephone.

Statistical analysis: The ASQ-3 was compared with the BSID-III across three domains; non-verbal, verbal and motor. The ASQ-3 and the BSID-III were also compared against the KBIT the using verbal and non-verbal domains. The cut-offs which normally trigger further investigation lie one standard deviation (SD) below the population mean (i.e. a score of less than 85) in the BSID-III and KBIT. These cut-off values were adjusted to one SD below the study cohort’s mean scores in each domain to ensure a geographically relevant cut-off and to account for the Flynn effect.^29^

For the purposes of our analyses, ASQ-3 scores were treated as pass or fail on each domain. An overall domain score landing in the black area which means that the child needs further assessment was deemed a referral trigger. These “fails” were compared with BSID-III which fell <1 SD below the cohort mean. A score in the grey zone which indicates that a child should be rescreened within six months was not considered a fail for the purposes of this study. When comparing the ASQ-3 and the BSID-III, the fine and gross motor domains for the ASQ-3 were combined to allow direct comparison to the BSID-III motor composite score. If a child triggered a referral for either fine motor, gross motor or both, then this was considered a referral for the purposes of comparison with the BSID-III motor composite score. The communication domain of the ASQ-3 was compared with the language domain of the BSID-III. Finally, the problem-solving score of the ASQ-3 was compared with the cognitive domain of the BSID-III. The problem-solving score (ASQ-3) and the cognitive domain (BSID-III) represent non-verbal for the purposes of our analyses and were compared with non-verbal scores for the KBIT.

The ASQ-3, the BSID-III and the KBIT were analysed for their levels of agreement and suitability as screening and assessment tools. Statistical analyses were completed using IBM SPSS Version 26 software. Descriptive statistics, Pearson correlations and concurrent validity and predictive ability were measured using sensitivity, specificity and area under receiver operating characteristic curve (AUROC). Sensitivity and specificity were used instead of positive and negative predictive value as this nested cohort of children who were small or thin for gestational age is at higher risk of developmental delay than the general population.

## Results

**Fig. 1:**
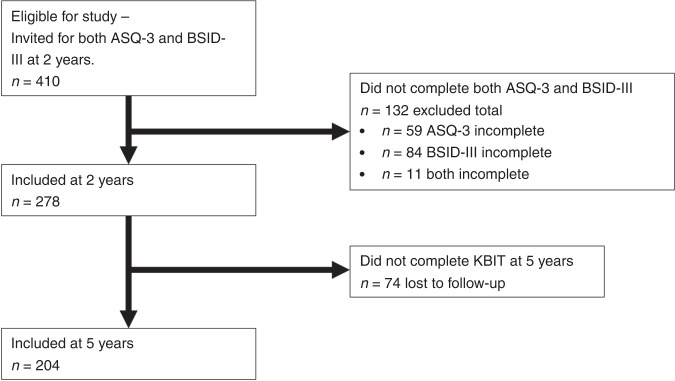
Study recruitment flow chart. Flow chart indicating cohort recruitment and assessment process including reasons for exclusion.

Four hundred and ten subjects were invited for both ASQ-3 and the BSID-III at 24 months. Participant flow chart and reasons for exclusion are outlined in Fig. 1. The mean (SD) birthweight of the children studied was 3445 (506) g, with a male-to-female ratio was 52:48. The mean(SD, range) for BW was 3445.58 (506.619, 3970), gestational age 39.56 (1.489, 13) weeks. Mean (SD) age assessment for ASQ and BSID-III respectively was 25.4 (1.4) and 26.9 (1.7) months. 13.5% (37/274, four values missing) were SGA. ASQ-3 and BSID-III assessments took place on the same day in 83.8% of cases, with some assessments taking 2–3 sessions to complete.

The subjects excluded due to non-completion of both assessments were compared with those who completed both (ASQ-3 and BSID-III). No significant differences were observed between the two groups (completed versus non-complete data groups) in their gestational age in weeks; sex distribution; maternal education; maternal marital status and maternal occupation. Figure 1 illustrates the attrition rate and numbers included at each stage of the study.

Outcome at 24 months: At 24 months, the mean(SD) BSID-III cognitive composite score of the cohort was 98.63 (11.30). The cohort specific cut-off of <1 SD was calculated as 87, with 29/278 (10.4%) children having a score below this cut-off. At 24 months, only 14/278(5%) children failed the problem-solving domain of the ASQ-3 including 8 false positives. For explanation of each domain’s geographically relevant cut-off (please see Table 1).

**Table 1:** Geographically relevant cut-off’s for BSID-III and KBIT.

Geographically relevant cut-off’s for BSID-III and KBIT			
	Mean	Standard Deviation (SD)	Mean < 1 SD
BSID-III Cognitive	98.63	11.30	87.33 (87)
BSID-III Language	106.65	15.29	91.36 (91)
BSID-III Motor	104.54	11.88	92.66 (92)
KBIT Verbal	107.34	9.54	97.8 (98)
KBIT Non-verbal	100.80	9.73	91.07 (91)

Cut-off scores based on 1SD below the cohort mean performance in each domain.

The concurrent validity data of the ASQ-3 and BSID-III at 24 months are displayed on Table 2. Of the 29/278(10.4%) children who had a BSID-III cognitive score <1 SD at 24 months, only 6/29 also had a fail score on the problem-solving domain of the ASQ-3 giving a sensitivity for the detection of cognitive delay of 20.7%. The ability of the ASQ-3 domains to detect language or motor delay was higher, with sensitivities of 25% and 50%, respectively. Specificities for cognitive, communication and motor domains were 96.8%, 99.5% and 76.1%, respectively. Overall predictive ability is displayed in Table 3. Again, whilst the motor and language domains of the ASQ-3 showed a reasonable predictive ability for a motor score <1 SD on the BSID-III, the predictive ability of the problem-solving domain for the cognitive scale of the BSID-III was poor.

**Table 2:** Concurrent validity of the ASQ-3 domain-specific clinical cut-offs and the language, motor and cognitive domains respectively of the Bayley Scales of Infant Development Version III (BSID-III).

	N	True Neg Pass both	False neg Pass ASQ-3, <1SD BSID	False Pos Clinical range ASQ-3/ Pass BSID	True Pos Clinical range ASQ-3/<1SD BSID
Cognitive	278	241	23	8	6
Communication	278	233	33	1	11
Motor	278	181	20	57	20

**Table 3:** Comparison of domain-specific sensitivities and specificities for ASQ-3 to detect an abnormal BSID-III (<1SD cohort mean).

		BSID-III relevant domain < 1SD cohort mean				
ASQ-3	*N*	Sensitivity	Specificity	AUROC	Range	*p* value
Cognitive	278	20.7%	96.8%	0.587	(0.467–0.708)	0.124
Communication	278	25%	99.5%	0.623	(0.521–0.724)	0.010
Motor	278	50%	76.1%	0.630	(0.532–0.728)	0.008

The overall predictive ability of the ASQ-3 differed with severity of delay (Table 4). For the 55 children with a BSID-III score <1 SD in any domain, 25 (45.5%) also failed at least one element of the ASQ. For the 13 with more severe delay (<2 SD) on any domain of the BSID-III, 11(84.6%) also failed at least one element of the ASQ.

**Table 4:** Ability of ASQ-3 to detect an abnormal BSID-III at 24 months with stratification for type of delay based on all-domain performance (Cognitive, communication and motor).

	BSID-III (all domain performance)			
	Severe (<2SD)	Mild-Moderate (<1SD)	Expected	Total
ASQ-3				
Fail Any Domain	11 (84.6%)	25 (45.5%)	46 (21.9%)	**80**
Pass All Domains	2 (15.4%)	30 (54.5%)	164 (78.1%)	**198**
Total	**13**	**55**	**210**	**278**

Mild-moderate delay is characterised as between 1SD and 2SD below the cohort mean. Severe delay is characterised by <2SD below the cohort mean.

Lastly, we examined the ability of both the ASQ and BSID-III at 24 months to predict cognitive difficulties at 5 years. Defining low average IQ as <1 SD for either verbal or non-verbal IQ on the KBIQ, 36/204(17.6%) had a low average verbal KBIT score and 24/204(11.8%) had a low average non-verbal KBIT score. The KBIT IQ composite score n, mean, (SD) = 204, 105.14, (8.88). The correlation between the BSID-III domains and KBIT scores at 5 years is displayed in Table 5. The cognitive composite score of the BSID-III correlated with both the verbal and non-verbal scores, and the Total IQ composite score at 5 years. The best correlation was seen between the language composite score at two years and the verbal score at 5 years. The ability of a low average ASQ and BSID-III to predict a low average IQ at 5 years is depicted in Tables 6 and 7. In all cases, high specificity was offset by low sensitivity. The best predictor of overall IQ at 5 years was the composite language score of the BSID-III at 2 years (Table 6).

**Table 5:** Correlation between Bayley Scales of Infant development-Version III (BSID-III) assessment at 2 years and Kaufmann Brief IQ test at 5 years.

BSID-III vs KBIT		Total verbal standard score	Total non-verbal standard score	IQ composite score
Language composite score	**R** (*p* value)	0.435 *P* < 0.001	0.158 *P* = 0.024	0.365 *P* < 0.001
Motor composite score	**R** (*p* value)	0.206 *P* = 0.003	0.110 *P* = 0.117	0.207 *P* = 0.003
Cognitive composite score	**R** (*p* value)	0.388 *P* < 0.001	0.342 *P* < 0.001	0.463 *P* < 0.001

*R* = Pearson’s correlation coefficient.

**Table 6:** Ability of ASQ-3 and BSID-III to predict <1SD below cohort mean performance in KBIT at 5 years of age in both verbal and non-verbal domains.

	AUROC (95% CI)	*p* value	Sensitivity	Specificity
Prediction of verbal IQ < 1SD in KBIT				
ASQ-3 communication	0.536(0.428–0.644)	0.502	8.3%	98.8%
BSID-III language	0.723(0.63–0.81)	<0.001	22.2%	88.7%
Prediction of non-verbal IQ < 1SD in KBIT				
ASQ-3 problem solving	0.528 (0.400–0.655)	0.659	8.3%	97.2%
BSID-III cognitive	0.695(0.59–0.80)	0.002	20.8%	94.4%
Prediction of overall IQ < 1SD in KBIT				
BSID-III language	0.732(0.64–0.82)	<0.001	15.6%	94.2%
BSID-III cognitive	0.696(0.61–0.79)	<0.001	18.8%	94.8%

**Table 7:** Domain-specific ability of ASQ-3 and BSID-III to predict <1SD below cohort mean performance in KBIT composite score at 5 years of age.

Prediction of KBIT composite score < 1SD		
	Sensitivity	Specificity
ASQ-3 problem-solving	12.5%	98.2%
BSID-III cognitive	15.6%	94.2%
ASQ-3 communication	9.4%	98.8%
BSID-III language	21.9%	88.4%
ASQ-3 motor	25%	75%
BSID-III motor	28.1%	88.4%

## Discussion

We have shown that the ASQ-3 has poor sensitivity for detecting mild-moderate delay when compared with the BSID-III at 24 months. Both the ASQ-3 and BSID-III demonstrate poor ability to predict low average cognitive outcomes at school age. However, BSID-III has slightly better sensitivities and significant AUROC’s whereas ASQ-3’s AUROCs are not statistically significant. The motor and language domains of the ASQ-3 performed best, whilst the cognitive domain showed the lowest concurrent validity and predictive ability at both time-points. The specificity of the ASQ-3 was high in all domains. This confirms the ability of the ASQ-3 to identify normative development, but also highlights that many children with cognitive difficulties at school age will be missed in countries relying on the ASQ-3 as a screening tool. Similar profiles of low sensitivity and high specificity have been reported for the ASQ-2 when compared with the Bayley Scales.^19^

The ASQ is one of the most widely used screening assessments of early child development, forming part of the recommended screening schedule in many countries, including the public health assessment protocol in Ireland, where this study was developed.^30^ The ease of administration, requiring care-giver report only, makes it attractive for widespread screening, particularly in low-resource countries. Adequate reliability and validity of the ASQ have been demonstrated in U.S. populations and other high-resource countries.^31^ Validity reported by the developers of the ASQ has used comparisons to early intervention/early childhood special education eligibility evaluations using the Battelle Developmental Inventory in high-risk groups referred for further assessment.^14^ These groups consisted of more than 50% deemed eligible for special education, making it difficult to compare this to the widespread use of the ASQ as a developmental screener in a healthy population. Data on the predictive ability of the ASQ has been reported in a number of countries, using locally adjusted versions of the ASQ in non-English speaking cohorts with calculated ROC ranging from 0.66 to 0.87.^32^ Data in English-speaking populations are limited with a systematic review having identified 32 publications related to this from a combined ten cohorts; of these ten cohorts only one used the English language ASQ.^33^

Although the ASQ is designed to give a global impression of the child’s development, the assessment is divided into individual domains. Our focus for this study was the prediction of cognitive delay. Whilst language and motor assessment concurrent validity between the ASQ and the BSID-III was more robust, detection of mild-moderate cognitive delay was very poor. We have also shown that the ASQ-3 is more effective at detecting severe delay than mild or moderate delay, in keeping with previous reports from Sheldrick et al.^34^ and Gollenberg et al.^18^ . When one looks at all-domain performance, 84.6% of those classified by the BSID-III as having severe delay were detected by the ASQ-3. Only 45.5% were detected in the mild to moderate category. Often, children with severe delay will likely be detected on routine observation by parents and medical professionals without the requirement for validated screening tools. Most children with cognitive delay fall into the mild to moderate^34^ category which is the cohort least likely to be detected by the ASQ-3 alone. These children are also the group who may benefit most from early detection and implementation of early intervention services.

This study focused on assessing the ASQ-3’s performance at two years, as recommended in Ireland’s national public health screening schedule, and prediction of cognitive outcomes at school-age. The communication and problem-solving domains of the ASQ-3 were assessed for their ability to predict future performance in the verbal, non-verbal and total intelligence domains of the KBIT respectively. The incidence of delay was similar to that seen in the total BASELINE cohort at 5 years.^27,35^ It should be noted that although KBIT is an abbreviated IQ assessment and is not a comprehensive test. It has demonstrated good reliability in assessing cognitive outcomes at school age compared to formal testing using either WISC-IV General Ability Index or the WAIS-III. It has been used in a number of large birth cohorts due to its ease of administration. It does not however give a detailed cognitive profile allowing separation of individual cognitive functions. Nor does it predict specific learning disabilities.^36–38^ Whilst the ASQ-3 showed excellent specificity, the sensitivity for the detection of low average non-verbal IQ was very low. This raises questions regarding the utility of the ASQ-3 as a screening tool for cognitive delay. We require screening tools to identify children suitable for further testing. As it is currently scored, the ASQ-3 has such high specificity that further testing is almost not required, whilst its low sensitivity means that the majority of cases of cognitive delay will be missed.

The BSID-III performed better than the ASQ-3 for prediction of outcome at 5 years. This is to be expected with a directly administered test. The overall prediction of both verbal and non-verbal IQ was acceptable, with AUROCs between 0.695 and 0.723. However, this was driven by very high specificity, meaning that the majority of children with low average IQ scores at 5 years were not detected by a BSID-III at 24 months. It is estimated that less than half of children with developmental delay are detected prior to school entrance, with the vast majority of those detected receiving no intervention in the very early years.^39,40^ Our study supports the need for better early assessments of cognitive ability and executive function to allow useful intervention and support prior to school entry. Both assessments were performed once at 24 months and it may be that repeated measures to assess the child’s trajectory over time may improve prediction.^41^

It is not surprising that the ASQ assessments based on caregiver report perform better in the quantification of tangible skills such as speech and motor milestones. As with all parentally reported assessments, there is an inherent bias, which may be influenced by the parents own socio-economic background, parenting experience or education level. Care-givers assessment of a child’s early problem-solving skills is inherently more difficult. However, it is these early executive functions which are most highly predictive of the lifetime course, opportunities and health of that child.^1^ We must recognise that current screening, whether by caregiver report or direct developmental assessment does not detect the majority of cases of low average cognitive ability. New methods of early assessment are required if we are to give these children opportunities to reach their full potential. This particularly applies to mild-moderate delay where early intervention may improve that child’s academic attainment and consequently their future quality of life.^42–46^

We have focused on examining the agreement between neurodevelopmental performance at 24 months and subsequent performance at school age as data on predictive performance is scarce.^33^ This study utilised BSID-III as the gold standard at two years of age. It should be noted that BSID-IV was released in 2019. It should be noted that the Bayley Scales of Infant and Toddler Development IV did not include the high-risk populations in the normative group as was the case for the BSID-III. Outcomes at five years of age will not be available with the BSID-IV for several years. The BSID-IV has updated its administrative protocol for ease of use and to shorten the assessment, without altering expected scores. Thus we feel that our findings are relevant for current use of either BSID-III or BSID-IV.^47^ There were some incomplete data in this study as patients were excluded due to non-completion of either the ASQ-3, the BSID-III or both. Subsequently, 74 further patients did not complete the school-age assessment at five years. However, the children with incomplete data did not differ from the group in their demographic or socioeconomic variables. The amount of time taken to complete the BSID-III may be a barrier to its completion with 45/278 requiring more than one session to complete the assessment. Each session may take 60–90 min. However, each subject was afforded the opportunity of multiple sessions to complete the BSID-III assessment. Our study was not focused on examining the acceptability or ease of use of both methods of assessment, but instead a direct comparison of detection of domain-specific delay.

## Conclusion

In summary, the parentally completed ASQ-3 has poor concurrent validity with the cognitive scales of the BSID-III at the same age. It is more effective at identifying children with severe developmental delay but does not achieve sensitivities requisite with an effective screening tool. Both the ASQ-3 and the BSID-III demonstrate poor ability to predict cognitive ability at school-age in both verbal and non-verbal domains. Both tests have high specificity and are adept at predicting a normal performance at 5 years. The majority of children with low average IQ at school age will not be detected using current screening methods.

## Data Availability

De-identified individual participant data (including data dictionaries) will be made available, in addition to study protocols, the statistical analysis plan, and the informed consent form. The data will be made available upon publication to researchers who provide a methodologically sound proposal for use in achieving the goals of the approved proposal. Proposals should be submitted to d.murray@ucc.ie
